# Periodic injections of adipose-derived stem cell sheets attenuate osteoarthritis progression in an experimental rabbit model

**DOI:** 10.1186/s12891-020-03718-z

**Published:** 2020-10-19

**Authors:** Tomoharu Takagi, Tamon Kabata, Katsuhiro Hayashi, Xiang Fang, Yoshitomo Kajino, Daisuke Inoue, Takaaki Ohmori, Takuro Ueno, Junya Yoshitani, Ken Ueoka, Yuki Yamamuro, Hiroyuki Tsuchiya

**Affiliations:** Department of Orthopaedic Surgery, Graduate School of Medical Sciences, Kanazawa University, Kanazawa university, 13-1 Takara-machi, Kanazawa, 920-8641 Japan

**Keywords:** Adipose-derived stem cells, Cell sheet, Osteoarthritis

## Abstract

**Background:**

Subcutaneous adipose tissue represents an abundant source of multipotent adult stem cells named as Adipose-derived stem cells (ADSCs). With a cell sheet approach, ADSCs survive longer, and can be delivered in large quantities. We investigated whether intra-articular ADSC sheets attenuated osteoarthritis (OA) progression in a rabbit anterior cruciate ligament transection (ACLT) model.

**Methods:**

Fabricating medium containing ascorbate-2-phosphate was used to enhance collagen protein secretion by the ADSCs to make ADSC sheets. At 4 weeks after ACLT, autologous ADSC sheets were injected intra-articularly into the right knee (ADSC sheets group), and autologous cell death sheets treated by liquid nitrogen were injected into the left knee (control group). Subsequent injections were administered once weekly. Femoral condyles were compared macroscopically and histologically.

**Results:**

Macroscopically, OA progression was significantly milder in the ADSC sheets than in the control groups. Histologically, control knees showed obvious erosions in the medial and lateral condyles, while cartilage was retained predominantly in the ADSC sheets group. Immunohistochemically, MMP-1, MMP-13, ADAMTS-4 were less expressive in the ADSC sheets than in the control groups.

**Conclusions:**

Periodic ADSC sheets injections inhibited articular cartilage degeneration without inducing any adverse effects. A large quantity of autologous ADSCs delivered by cell sheets homed to the synovium and protected chondrocytes.

## Background

Osteoarthritis (OA) is characterized by articular cartilage destruction along with changes occurring in other joint components including bone, synovium, ligaments, and menisci [1, 2]. Although surgical interventions, such as joint arthroplasty and osteotomies, have been pursued in many patients to relieve joint pain and improve joint function, most OA patients are managed conservatively with oral or topical medications, intra-articular corticosteroids or hyaluronic acid, and physiotherapy [3, 4]. However, these approaches focus only on temporal improvement of the symptoms rather than treating the complicated pathogenesis of OA.

Mesenchymal stem cells (MSCs), capable of self-renewing and multi-lineage differentiation, are considered to have great potential for application in regenerative medicine. Among the various sources of MSCs, adipose-derived stem cells (ADSCs) represent an abundant source of multipotent adult stem cells that are easily isolated from subcutaneous adipose tissue and cultured in large quantities with a minimally invasive procedure [5, 6]. It is thought that 1% of nucleated cells in adipose tissue are ADSCs, whereas only 0.001–0.002% of nucleated cells in bone marrow, which is currently a common source of MSCs, are stem cells [7]. In addition to the ability of self-renewal, ADSCs can differentiate into multiple lineages when cultivated under specific induction conditions, including osteogenic, chondrogenic, adipogenic, and myogenic lineages [8]. This ability, together with their easy accessibility and low donor site morbidity, has made ADSCs good candidates for a broad range of cell-based therapeutics. Therefore, we investigated them in various fields such as cartilages, bones, peripheral nerves, ligaments, and menisci [5, 9–12].

Recently, cell sheet technology has been applied to enhance the regenerative effects of tissue-engineered [13]. With a cell sheet approach, cells expanded in sheets are harvested together with their autocrine extracellular matrix (ECM) and intact cell-cell connections [14]. Application of ADSC-based cell sheet technology has been successful in creating tissue-engineered adipose substitute, treatment of chronic heart failure and enhanced skin wound healing [13, 15, 16]. Although there are few reports that ADSCs attenuate inflammation in OA, the role of ADSC sheets in the treatment of OA and the regenerative mechanism are not understood well. Therefore, we determined whether intra-articular ADSC sheets inhibited articular cartilage destruction during OA development in a rabbit anterior cruciate ligament transection (ACLT)-induced OA model; in particular we focused on the possibility that more ADSCs survive with cell sheets transplantation.

## Methods

### Experimental animals

Twenty-six skeletally mature female Japanese white rabbits (age: 6 months old, body weight: 2.5–3.0 kg, KITAYAMA LABES, Nagano, Japan) were used for this experiment. The Institute for Experimental Animals, Kanazawa University Advanced Science Research Center approved all experimental protocols, using an animal model. All surgical procedures were performed in accordance with the Guide for the Care and Use of Laboratory Animals published by the United States National Institutes of Health (Bethesda, MD; NIH publication no.86–23, revised 1985).

### Induction of experimental OA

**Fig. 1:**
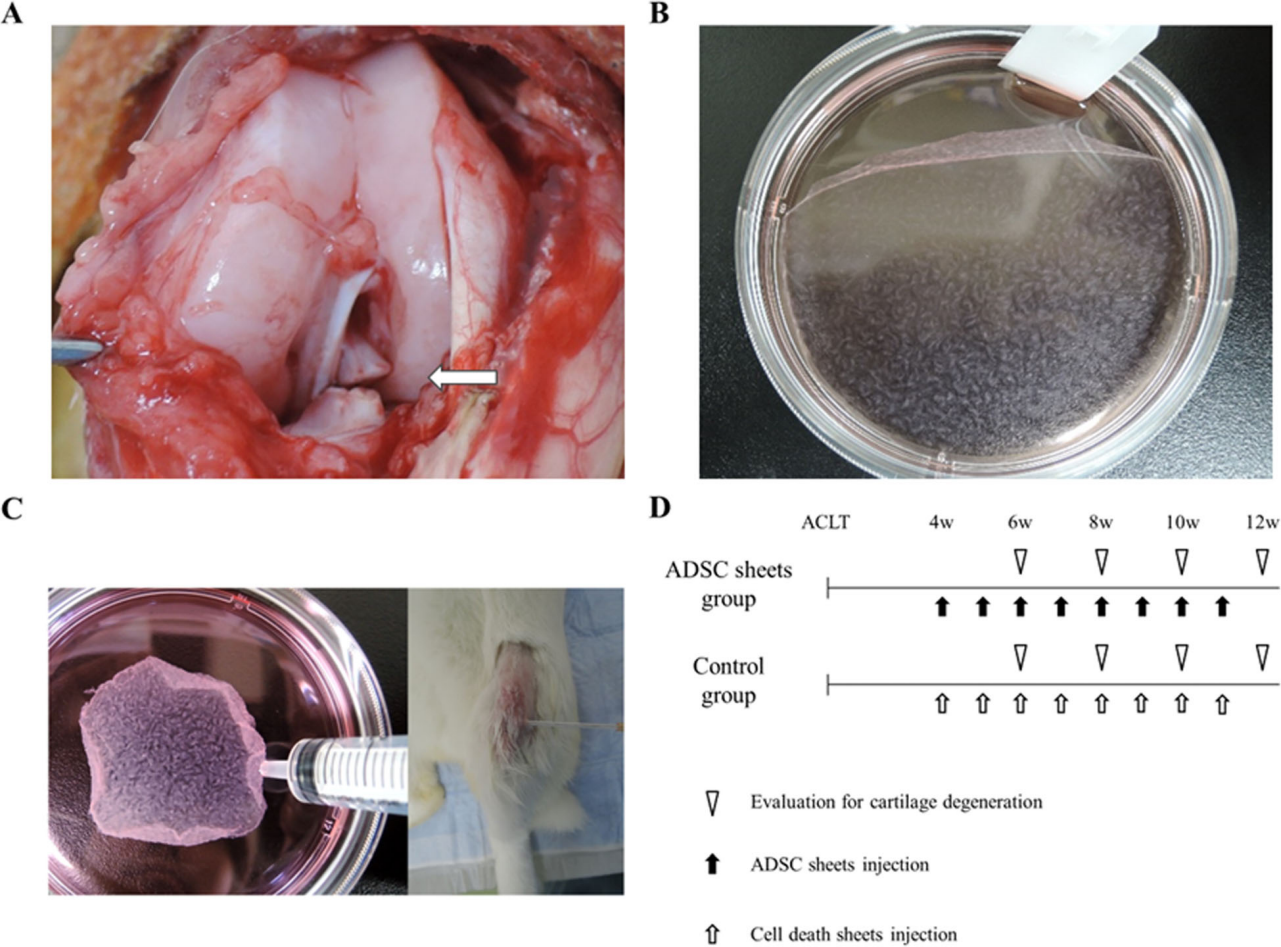
**a** Anterior cruciate ligament transection is indicated by white arrows. **b** ADSCs were detached as sheets. **c** ADSC sheets in 1 mL PBS were injected intra-articularly, using 14-gage needles. **d** Schema of the study

Induction of OA was performed as described in previous study [17]. The rabbits were anesthetized by intramuscular injection of ketamine hydrochloride (35 mg/kg) and xylazine (5 mg/kg), and intravenous injection of pentobarbital sodium (50 mg/kg). Both knees were shaved and sterilized with iodine. The right knee joint was exposed through a medial parapatellar incision. The patella was dislocated laterally, and the knee was placed in full flexion. The ACL was visualized optimally and transected with a micro-scissor (Fig. 1a). A positive anterior drawing test was performed to confirm complete ligament transection. The joint was irrigated and sutured in a routine fashion. An identical operation was performed in the left knee. Postoperatively, free activity was allowed in the cage without immobilization. Traumatic degeneration was induced as described previously for the ACLT model [18], which is characterized by OA-like damage. The rabbits showed considerable individual variability in OA progression at 4 weeks after ACLT [19]. Therefore, we used matched-pair analysis to examine the chondroprotective effect of ADSC sheets on OA progression in a stricter manner.

### Isolation of ADSCs

ADSCs were isolated by a previously reported method [5]. Adipose tissue (approximately 1–2 g) was harvested from the posterior neck regions of the rabbits and washed with phosphate-buffered saline (PBS; Wako Pure Chemical Industries, Osaka, Japan). The tissue was cut into strips over 5 min. Collagenase (Wako Pure Chemical Industries) was dissolved in PBS to a concentration of 0.12% in 25 mL and used to digest adipose tissue at 37 °C for 45 min in a water bath. The mixture was shaken every 15 min during the digestion period. Immediately after the reaction was completed, 25 mL of Dulbecco’s modified Eagle’s medium (DMEM; Wako Pure Chemical Industries), was added to neutralize collagenase activity. The resulting solution was filtered and centrifuged at 1300 rpm (rpm) for 6 min at 25 °C, and the supernatant was removed. Next, a pellet of cells was seeded at 5 × 10^4^ cells/cm^2^ in 100 mm tissue culture dishes and cultured in DMEM containing 10% fetal bovine serum (FBS; Nichirei Biosciences, Tokyo, Japan) and 1% Penicillin-Streptomycin Solution (P/S; Wako Pure Chemical Industries) at 37 °C in 5% CO_2_ incubator. After 24 h, debris was removed by washing with PBS and fresh medium was added. Plastic-adherent spindle-shaped cells became apparent 3–4 days later and were isolated with trypsinization after reaching 80–90% confluence. More than three passage ADSCs were prepared for the subsequent experiment. Previous reports have confirmed the presence of stemness in ADSCs extracted by this method [6, 20].

### Fabrication of ADSC sheets

ADSC sheets were fabricated as reported previously [21]. To create cell sheets, ADSCs were seeded in the 100 mm tissue culture dishes by 1 × 10^6^ cells/dish for 7 days. The culture medium consisted of DMEM, 10% FBS, 1% P/S, and 50 mM ascorbate-2-phosphate (vitamin C [Vc]). The culture medium was refreshed every 2–3 days in 1 week. The fabricating medium containing ascorbate-2-phosphate was used to enhance the collagen protein secretion by the ADSCs to make the ADSC sheets (Fig. 1b).

### Injection of ADSC sheets

Twenty-four rabbits (48 knees) were used for matched-pair analysis. Autologous ADSC sheets were fabricated from the subcutaneous adipose tissue parallel to induction of experimental OA. ADSC sheets were rinsed with PBS three times. Autologous cell death sheets were made for control by liquid nitrogen according to a previous report [22]. At 4 weeks after ACLT, autologous ADSC sheets in 1 mL PBS were injected intra-articularly into the right knee (ADSC sheets group), using 14-gage needles (Fig. 1c). Similarly, autologous cell death sheets were injected into the left knee (control group). Subsequent injections were administered once weekly. The rabbits were sacrificed following the intravenous injection of 6 ml sodium pentobarbital at 6, 8, 10 and 12 weeks after ACLT (6 rabbits in each week), and femoral condyles from both knees were harvested (Fig. 1d).

### Macroscopic analysis

To assess cartilage lesions, the femoral condyles were stained with India ink (American MasterTech, CA, USA). A Canon IXY 650 digital camera (Canon, Tokyo, Japan) was used to take macroscopic pictures. Gross findings were classified and scored as described previously [23, 24]. The medial and lateral femoral condyles were scored individually as grade 0, intact articular surface; grade 1, minimal fibrillation; grade 2, overt fibrillation; grade 3, erosion of 0–2 mm; grade 4, erosion of 2–5 mm; and grade 5, erosion of > 5 mm. The two scores were summed to obtain a cumulative macroscopic OA score. All evaluations were performed by two blinded researchers and their scores were averaged to obtain an overall score.

### Histologic analysis

The dissected distal femurs were fixed in a 4% paraformaldehyde solution after gross morphologic examination, and subsequently decalcified in 4% ethylenediaminetetraacetic acid (EDTA) solution. The specimens were dehydrated with a gradient ethanol series, embedded in paraffin blocks, and cut into 5-μm sections. Safranin O staining was used to assess general morphology and proteoglycans/collagen content in cartilage. Histologic sections were visualized using a fluorescence microscope (Keyence Japan, Osaka, Japan). Ten coronal sections were prepared in the coronal plane through the middle of the femoral condyles, and one section from each sample, which included the most severely degenerated area, was used for each histologic analysis. Two blinded investigators evaluated the severity of cartilage degeneration using the cartilage OA histopathology grading system methodology of the Osteoarthritis Research Society International (OARSI) [25].

### Immunohistochemical analysis

The analyses were performed as follows to evaluate cartilage matrix protein catabolic enzymes [26]. After deparaffinization, sections were incubated with 0.3% hydrogen peroxide for 30 min, and subsequently treated with hyaluronidase for 60 min. Then, they were incubated at room temperature with mouse anti-human MMP-1 monoclonal antibody (1:100; Kyowa Pharma Chemical Co. Toyama, Japan), mouse anti-rabbit MMP-13 monoclonal antibody (1:20; Thermo Fisher Scientific Inc. Waltham, USA), and mouse anti-human ADAMTS-4 monoclonal antibody (1:150; Thermo Fisher Scientific Inc. Waltham, USA) respectively. All antibody dilutions were made in PBS. After an overnight reaction with the primary antibody at 4 °C, sections were incubated with labeled polymer-HRP anti-mouse IgG (Dako, Tokyo, Japan) at room temperature for 30 min. Signals were visualized with 3, 3′-diaminobenzidine tetrahydrochloride, and nuclei were counterstained with hematoxylin. Six microscopic fields (× 100 magnification) relative to the medial, central and lateral regions in cartilage tissue were used to perform a semi-quantitative analysis of immunohistochemistry. A semi-quantitative method that assigns immunohistochemistry values as a percentage of positive cells (MMPs, ADAMTS-4) was provided for a complete assessment of protein expression, with maximum scoring being 100% [27]. Immunohistochemical analysis results were evaluated by two observers blinded to the identity of each sample.

### 1,10-dioctadecyl-3,3,30,30-tetramethylindocarbocyanine perchlorate (DiI) labeling

A DiI labeling examination was performed to monitor the fate of the ADSCs after the intra-articular injection. Two rabbits (four knees) were used for this examination. Experimental OA was induced and autologous ADSC sheets and autologous ADSCs were fabricated. At 4 weeks after ACLT, autologous ADSC sheets (2.0 × 10^6^ cells) were injected into the right knee, and ADSCs (2.0 × 10^6^ cells) were injected into the left knee. On the day of injection, ADSC sheets and ADSCs were labeled for cell tracking with a fluorescent lipophilic tracer, DiI (Molecular Probes, Eugene, OR, USA) as indicated by the manufacturer. The rabbits were sacrificed at 6 weeks after ACLT, and the femoral condyles were dissected. Fresh frozen sagittal sections containing a central portion of the knee joint were prepared using Kawamoto’s method [28].

### Statistical analysis

SPSS ver.23.0 (SPSS, Inc., Chicago, IL, USA) was used to perform statistical analysis. The results are shown as the mean ± standard deviation. A paired t-test was used to perform matched-pair analyses. *P* < 0.05 were considered significant.

## Results

### Macroscopic analysis

**Fig. 2:**
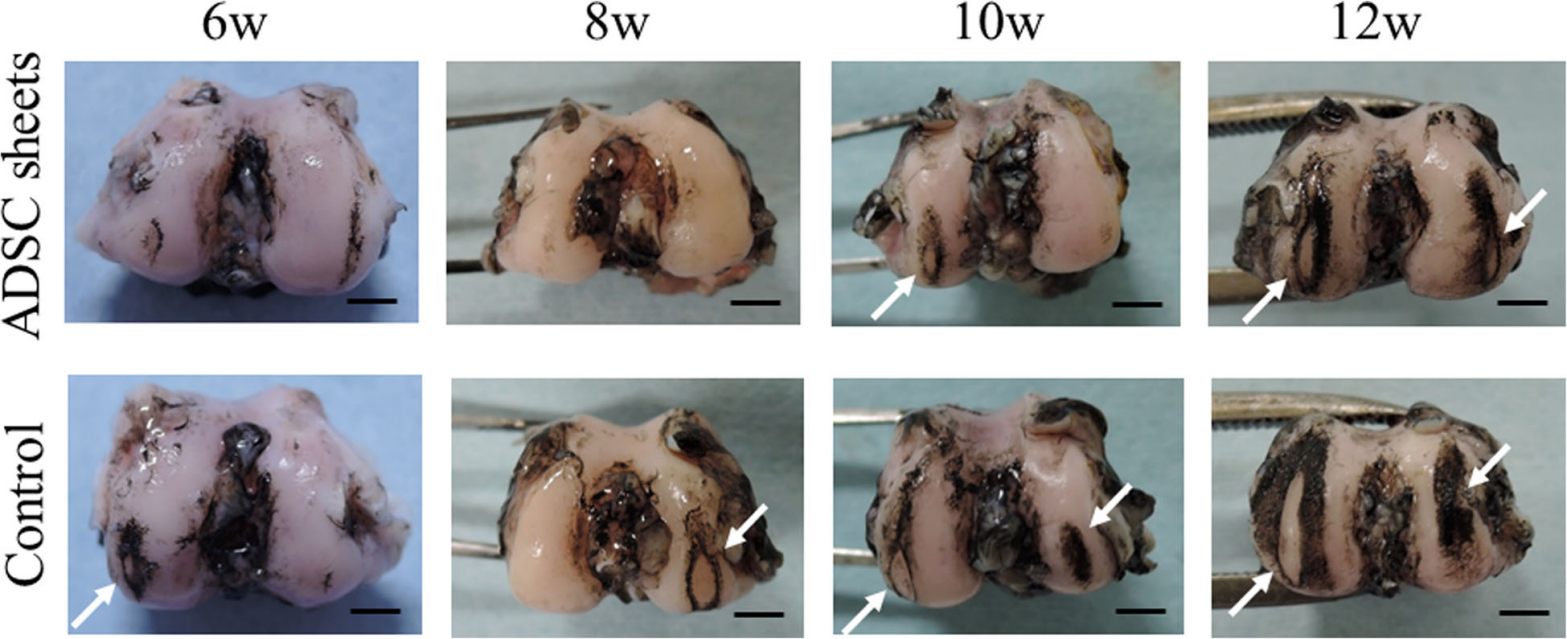
Representative macroscopic features of the femoral condyles stained with India ink. Cartilage erosion is indicated by white arrows. Scale bars = 5 mm

Figure 2 shows typical examples of control (cell death sheets) and ADSC sheets group knees stained with India ink. At 6 weeks after ACLT, mild erosion in the medial or lateral femoral condyle was observed in the control group, whereas near normal cartilage or fibrillation was observed in the ADSC sheets group. At 8 weeks, severe erosion was observed in the control knees. By contrast, only a few fibrillations were noted in the ADSC sheets group. At 10 weeks, erosions were observed in both groups, but the damaged areas in the ADSC sheets group were smaller than those in the control group. At 12 weeks, severe erosion in the medial and lateral femoral condyles was observed in both groups.

**Fig. 3:**
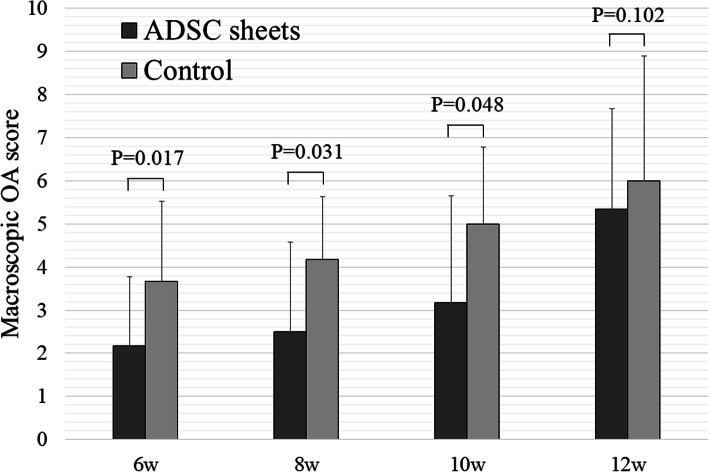
Macroscopic OA scores of ADSC sheets and control groups (*n* = 6)

Figure 3 shows the macroscopic OA scores. At 6, 8, and 10 weeks after ACLT, the mean scores of the ADSC sheets group were significantly lower than those of the control group (*P* < 0.05). These scores indicated less damage to the cartilage surface. At 12 weeks, the ADSC sheets group showed a slight suppression of cartilage degeneration unlike the control group, but there was no statistically significant difference (*P* = 0.102).

### Histologic analysis

**Fig. 4:**
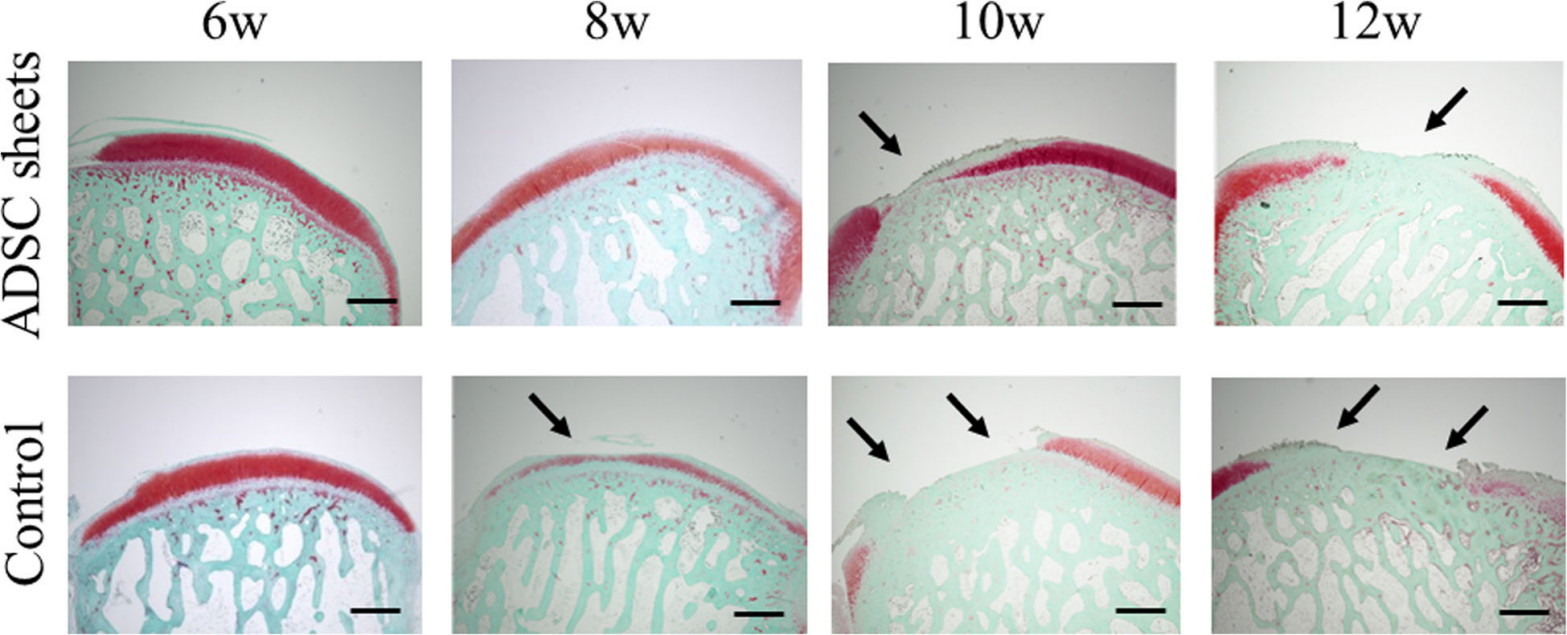
Representative specimens of the medial femoral condyles stained with Safranin-O. Cartilage erosion is indicated by black arrows. Scale bar = 1000 μm

**Fig. 5:**
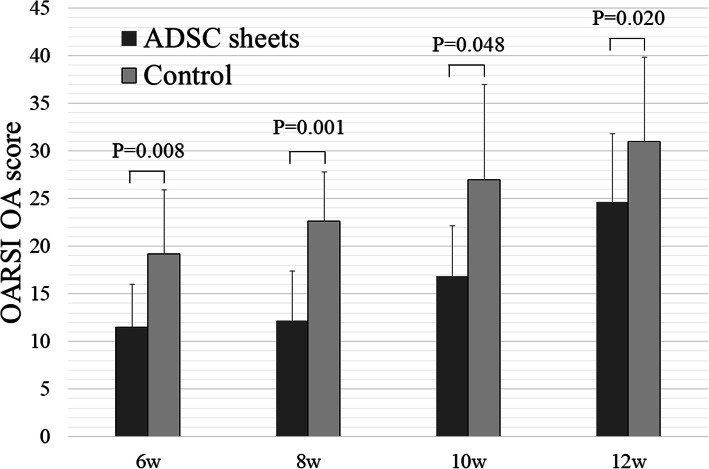
OARSI OA scores of ADSC sheets and control groups (n = 6)

Histologically, severe cartilage defects and decreased Safranin-O-staining intensity were observed in the control (cell death sheets) group, whereas fewer cartilage defects and decreased proteoglycan loss were observed in the ADSC sheets group at any time after ACLT (Fig. 4). Compared to the control group, ADSC sheets treatment significantly lowered the OARSI OA scores (*P* < 0.05; Fig. 5). At 12 weeks after ACLT, greater loss of cartilage was observed in both groups, but nevertheless ADSC sheets treatment revealed a significantly decreased OARSI OA score (*P* = 0.020). Histologic analyses demonstrated the protective role of ADSC sheets on the structure of cartilage tissue in the femoral condyle.

### Immunohistochemical analysis

Since degradation of cartilage matrix represents a key event in OA development, we tested to test the effect of ADSC sheets treatment on catabolic and inflammatory molecules involved in OA onset. MMP-1, MMP-13, and ADAMTS-4 are major proteases degrading the ECM. Expression of these enzymes was analyzed by immunohistochemistry using samples prepared 8 weeks after ACLT.

**Fig. 6:**
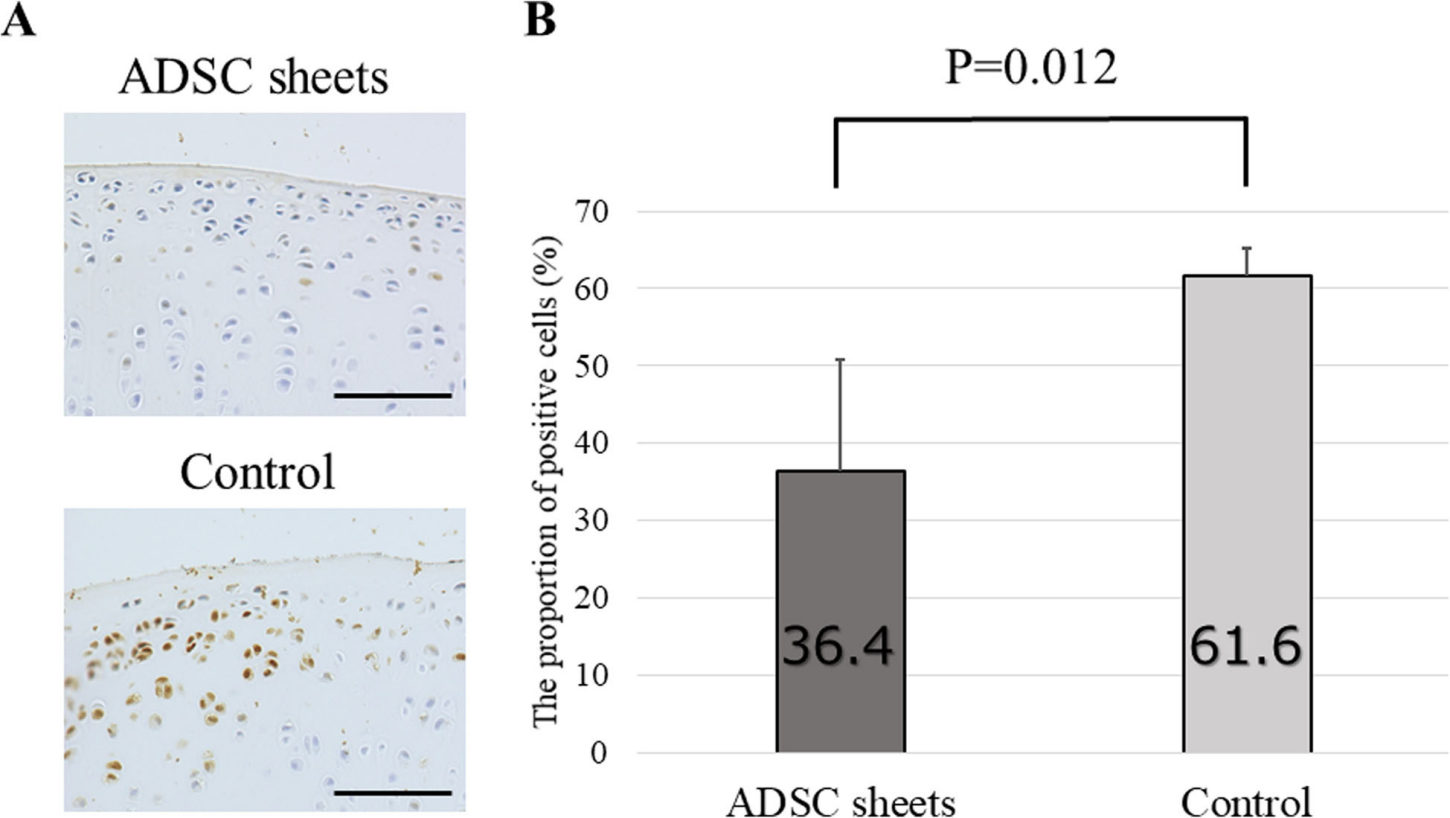
Immunohistochemical analysis for MMP-1. **a** Representative specimens 8 weeks after ACLT. Medial femoral condyles from the same individuals are shown. Brown staining = MMP-1 positive cells. Scale bars = 500 μm. **b** The proportion of MMP-1–positive cells (*n* = 6)

**Fig. 7:**
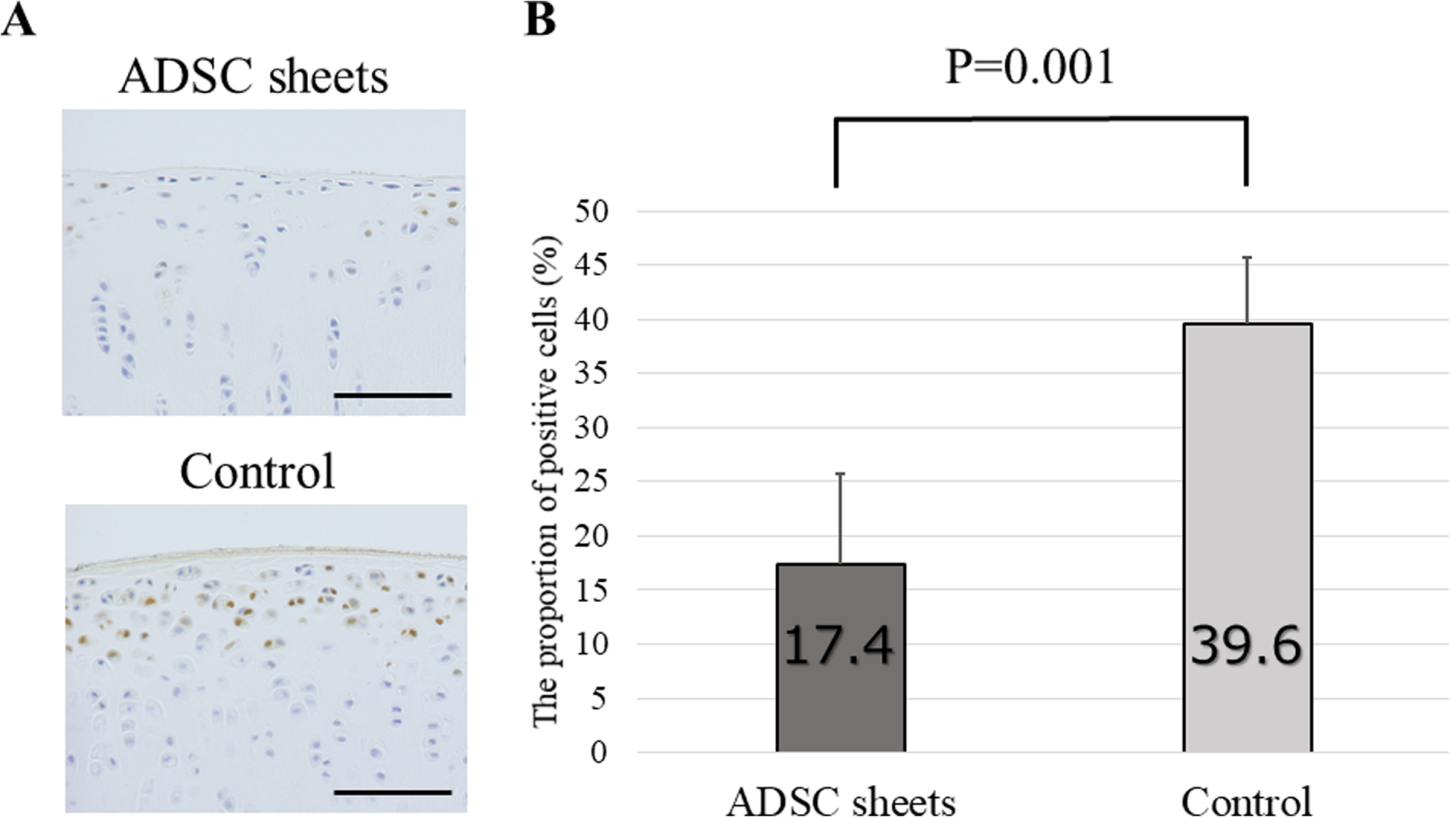
Immunohistochemical analysis for MMP-13. **a** Representative specimens 8 weeks after ACLT. Medial femoral condyles from the same individuals are shown. Brown staining = MMP-13–positive cells. Scale bars = 500 μm. **b** The proportion of MMP-13 positive cells (*n* = 6)

**Fig. 8:**
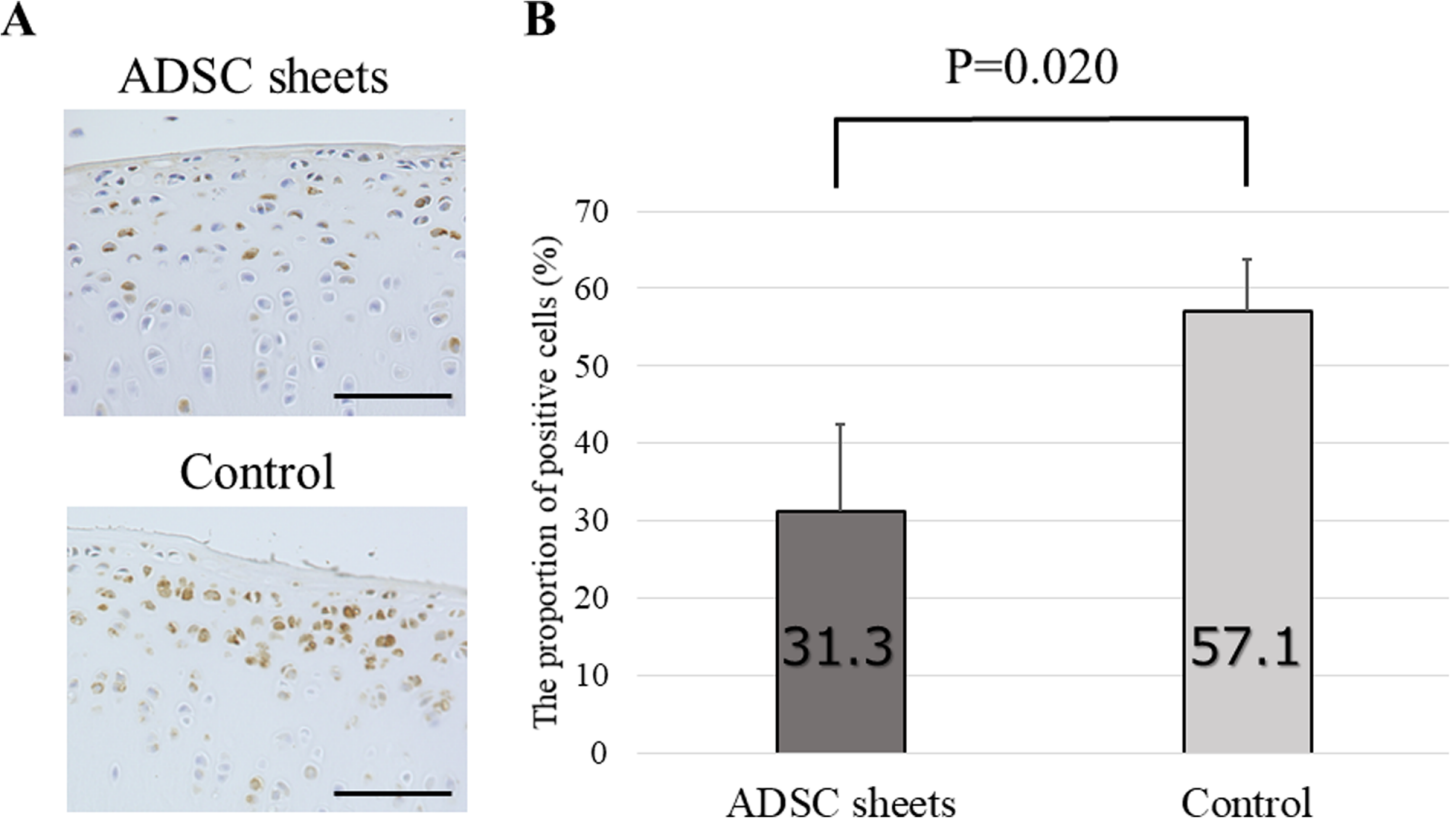
Immunohistochemical analysis for ADAMTS-4. **a** Representative specimens 8 weeks after ACLT. Medial femoral condyles from the same individuals are shown. Brown staining = ADAMTS-4 positive cells. Scale bars = 500 μm. **b** The proportion of ADAMTS-4–positive cells (*n* = 6)

High percentages of MMP-1-positive areas were noted in the control (cell death sheets) group, particularly in the transitional to radial cartilage zones. On the other hand, decreased MMP-1 was detected in the ADSC sheets group (*P* = 0.012; Fig. 6). For MMP-13, the control group displayed a moderate positive area expression. By contrast, the ADSC sheets-treated group showed low expression of MMP-13 compared to the control group (*P* = 0.001; Fig. 7). Equally, the proportion of ADAMTS-4 positive cells was significantly lower in sections of the ADSC sheets groups than in sections of the control group (*P* = 0.020; Fig. 8).

## DiI labeling

**Fig. 9:**
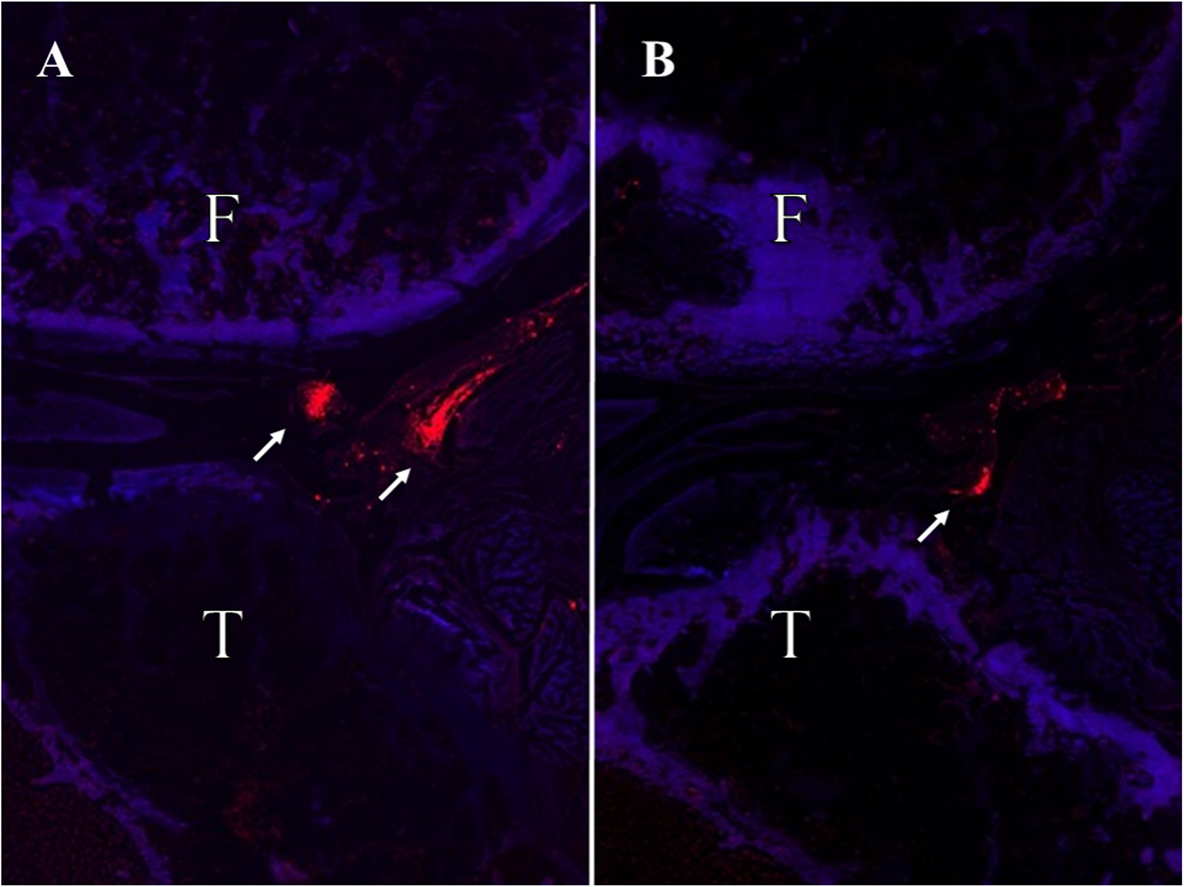
Biodistribution of DiI labeled ADSC sheets **a** and DiI labeled ADSCs **b** at 6 weeks after ACLT under epi-fluorescence. DiI positive cells were clearly detected in the subintimal layers of the synovium. Red staining = DiI positive cells. F, Femur; T, Tibia

Figure 9 shows DiI-positive areas. Intra-articularly injected ADSCs survived and homed to the lining layer of synovial membrane. More ADSCs survived in the knees transplanted with ADSC sheets (Fig. 9a). No cell engraftment was observed in the articular cartilage from the femoral condyle of any specimen evaluated.

## Discussion

Cell sheet engineering was developed as an advanced approach, designed to avoid the shortcomings of traditional tissue engineering. Studies have shown that cell sheets have biological scaffolds composed of collagen type I and can be transplanted with intact cell–cell junctions and undamaged ECM [16, 29]. Therefore, they increase survival of cells [30], enable the delivery of large number of cells [15], and enhance the stemness and transdifferentiation capability [31]. Presently, several improvements have been made to harvest the living cell sheet more easily, such as a temperature-responsive culture dish [32], the coating of dishes with a thermo-responsive hydrogel [33], and so forth. However, the entire grafting process remains relatively complicated, time-consuming, and requires special materials. In our study, a simple and inexpensive Vc-mediated procedure was used to obtain ADSC sheets [20].

There are several reports on the characteristics of Vc-induced cell sheets. Ando et al. [34] reported that Vc-induced cell sheets formation derived from synovial MSCs possessed sufficiently self-supporting mechanical properties despite not containing artificial scaffolding. Additionally, they revealed that the sheets contained collagen I and III, fibronectin, and vitronectin, and exhibited stable adhesion to the surface of cartilage matrix. We investigated the optimal condition of Vc-induced ADSC sheets formation and indicated osteogenic potential of the sheets^20^. However, the properties of Vc-induced cell sheets have many unclear parts such as strength, species, incubation period, and quantity of Vc. The Vc-induced ADSC sheets used in this study had the property of transplanting many ADSCs alive and was effective in suppressing articular cartilage degeneration. However, there may be better conditions for fabrication of them. Therefore, further studies are needed to decide an appropriate dosage of Vc that could make the cell sheets reach the balance point of transdifferentiation capability and mechanical strength.

Stem-cell therapy is an emerging treatment option for OA. Inspection by animal experiments is required to establish the new treatment, but it is necessary to use animal models with good reproducibility that are suitable for an experiment on this occasion. We evaluated the efficacy of intra-articular injection of autologous ADSC sheets to treat OA in an experimental rabbit model. We chose a rabbit OA model induced by ACLT surgery, because the ACLT model is similar to the pathology of human OA and has been widely validated for investigating OA [5, 17, 24]. Additionally, the mouse and rat models are too small to inject the cell sheets we made, and making the OA knee is difficult in the pig or large animals. Furthermore, it is difficult to secure a population. The rabbit was the most appropriate for this experiment due to its experimental ease. However, the rabbit does not meet the characteristic of the human knee in that it is not bipedal. Inspection in bipedal animals, such as a chicken or monkey, is desirable in the future.

Previous studies have evaluated the safety and efficacy of ADSCs for OA treatment. Desando et al. [27] found that autologous ADSCs attenuated inflammation in synovial membranes and prevented damage to cartilage and menisci in a rabbit ACLT model. Kuroda et al. [5] revealed that a few DiI-ADSCs homed to intra-articular soft tissue and showed the trophic effects in a rabbit OA model. In this study, a large quantity of DiI-ADSCs became clumped and survived in the subintimal layers of the synovium and protected chondrocytes from inflammatory factor-induced damage. There was no evidence of local inflammation. We did not find the formation of neocartilage in both groups, but the OA progression was significantly milder in the ADSC sheets group. After ACLT, as time progressed, more serious OA developed in both groups. However, the cartilage was thicker in the ADSC sheets group. To the best of our knowledge, our study is the first to prove the role of autologous ADSC sheets in the treatment of OA, and further research is needed to improve the effectiveness of ADSC sheets.

Some possible mechanisms of action underlie the effectiveness of MSCs in the OA treatment. First, injected MSCs differentiate into chondrocytes and fill articular cartilage lesions [35]. Second, injected MSCs could influence the microenvironment via trophic mechanism of action by the release of chondroprotective growth factors and cytokines [5]. Our data showed that MMP-1, MMP-13, and ADAMTS-4 were less expressive in the ADSC sheets group. MMPs comprise a large group of zinc-dependent proteases that can degrade components of the extracellular matrix such as collagen, elastin gelatin, and casein [36]. ADAMTS proteases are multidomain extracellular protease enzymes. Their functions include processing of procollagens as well as cleavage of aggrecan, versican, brevican and neurocan. MMPs and ADAMTS family have crucial roles in the initiation and progression of cartilage damage during OA. MMP-1 and MMP-13 degrade type II collagen, which is the main component of the cartilage matrix and is responsible for the degradation of native collagen fibers [37]. ADAMTS-4 is a major enzyme responsible for aggrecan degradation [38]. Therefore, we suggested that the ADSC sheets inhibited progression of articular cartilage degeneration by secreting liquid factors having chondroprotective effects. In short, we agree with the second mechanism.

At the early stage of ADSC sheets OA treatment, we suggested that the secretion of trophic factors by autologous ADSCs is the main mechanism responsible for their chondroprotective effect. Injection of ADSCs may be advantageous for secreting various factors to mediate anti-inflammatory, anti-fibrotic, and anti-apoptotic functions [39]. However, as time progresses, the trophic effect of them will become weaker. The OA knee model used in this study was induced by the instability of the joint caused by the dissection of the ACL. Since ADSCs could not suppress the mechanical factor, overt degeneration occurred in both groups at 12 weeks in this study. To maintain the chondroprotective effect, we suggested periodic injection of large number of autologous ADSCs, namely, weekly injection of ADSC sheets are needed. Still, complete suppression of OA progression would be impossible. Stem cell therapy of OA caused by the mechanical factor may play only an adjunct role in treatment. Further studies are necessary to prove this hypothesis.

Our study has some limitations. First, the long-term culture may have changed the properties of the ADSCs sheet at late phase injection. As mentioned above, there are many unclear points about the characteristics of the ADSC sheets, and the change with the culturing period and the optimal amount of Vc are the subject of future research. Second, the details of the ability of ADSCs to differentiate into chondrocytes are not known. The possible mechanism by which stem cells differentiate into chondrocytes and accumulate in lesions was discussed above. Although this study did not mention the mechanism, we believe that if ADSCs can be induced to differentiate into chondrocytes, further therapeutic effects can be expected. Third, we have not verified the effects of cell sheets. Specifically, the progress of OA was not evaluated between the ADSC sheets transplantation and the only ADSCs transplantation. However, it is technically difficult to transplant and retain the same number of cells as the ADSC sheets without creating the sheet. In the present study, as shown by the DiI labeling data, the cell sheets transplantation clearly had a greater amount of cell colonization in the joint than the cells alone transplantation. From this point, the cell sheet approach is considered to have great significance.

## Conclusions

Periodic injections of ADSC sheets attenuated OA progression in an experimental rabbit model without inducing any adverse effects. A large quantity of autologous ADSCs delivered by cell sheets homed to the synovium and protected chondrocytes.

## Data Availability

The datasets used and/or analysed during the current study are available from the corresponding author on reasonable request.

## Abbreviations

ADSCs: Adipose-derived stem cells
OA: Osteoarthritis
ACLT: Anterior cruciate ligament transection
MMP: Matrix metalloproteinase
ADAMTS: A disintegrin and metalloproteinase with thrombospondin motifs
MSCs: Mesenchymal stem cells
ECM: Extracellular matrix
PBS: Phosphate-buffered saline
DMEM: Dulbecco’s modified Eagle’s medium
FBS: Fetal bovine serum
Vc: Vitamin C
EDTA: Ethylenediaminetetraacetic acid
OARSI: Osteoarthritis Research Society International
DiI: *1,10-dioctadecyl-3,3,30,30-tetramethylindocarbocyanine perchlorate*
